# Editorial: Special Issue “Laser Synthesis and Processing of Nanostructured Materials”

**DOI:** 10.3390/nano14040344

**Published:** 2024-02-11

**Authors:** Oleg Vitrik, Aleksandr Kuchmizhak

**Affiliations:** 1 Institute of Automation and Control Processes (IACP), Far Eastern Branch of the Russian Academy of Science, Vladivostok 690091, Russia; oleg_vitrik@mail.ru; 2 Far Eastern Federal University, Vladivostok 690091, Russia

The fabrication of functional nanomaterials and nanotextured surfaces assisted by spatially and temporally confined laser radiation has matured from laboratory-scale methods to application-ready technology during recent decades. The interaction of intense pulsed laser radiation with matter can induce an intricate sequence of physical processes, including ultrafast phase transitions, thermomechanics and self-organization driven by hydrodynamic instabilities and/or interference effects. The diversity of laser-induced processes allows one to realize various efficient and scalable approaches for sculpting matter at the nanoscale, empowering it with new functions. Special Issue was focused on recent trends in the fabrication of functional nanostructures, nanomaterials and nanotextured surfaces using laser radiation. The resulting content of this Special Issue comprising 10 original papers reflects the diversity of the developing nanofabrication strategies that utilize pulsed laser radiation, including pulsed laser ablation in liquid (PLAL) for nanomaterial generation [1,2,3,4], pulsed laser deposition (PLD) of functional coatings [5], laser-induced forward transfer (LIFT) [6], laser sintering [7], pulsed laser surface nanotexturing [8] and direct laser writing in transparent media [9,10].

PLAL represents actively developing easy-to-implement laser-assisted technology for the production of functional nanomaterials [11,12,13,14,15]. The growing popularity of the method is evident from the analysis of this Special Issue content, where four published papers address PLAL. The technology resembles, to some extent, a kind of chemical synthesis, where the spatially and temporally confined laser radiation irradiates the bulk material target or nanoparticles placed in a liquid environment, driving the generation of nanomaterials and their modification through the localized chemical reactions at the liquid–solid interface. Specific synthesis conditions created by light (such as high temperatures and pressures, fast heating/quenching rates, etc.) allows one to produce a diversity of unique nanomaterials with sophisticated structure as well as a unique chemical/phase composition defined by laser synthesis conditions and the reactivity of the surrounding liquid. Hybrid nanomaterials (nanohybrids) composed of different elements properly arranged at the nanoscale to improve the basic nanomaterial functionality are the focus of many state-of-the-art studies on PLAL [16,17,18,19,20,21,22].

A perfect example of expanded functionality originating from a combination of different materials within unified nanoparticles was given by Bubnov et al., who reported on PLAL-based synthesis of silicon–iron (Si-Fe) nanohybrids [1]. In particular, the authors demonstrated that such a material combination is favorable for the spectrally strong broadband light-to-heat conversion performance of the produced nanoparticles, while the presence of Fe atoms provides them with magnetic properties. As a result, the produced nanohybrids hold promise for cancer theranostic applications, where such vital modalities as magnetic resonance imaging/guiding can be efficiently combined with photothermal therapy. In another example, Mincheva et al. reported the synthesis of two types of nanohybrids made by mixing titanium dioxide (TiO_2_) and zinc oxide (ZnO) [2]. Such complex hybrid nanomaterials were obtained by sequential ablation of Ti or Zn bulk targets in distilled water by millisecond laser pulses and demonstrated superior photocatalytic and chemiresistive gas sensing performance toward volatile organics in comparison with single-metal oxides. Moreover, the authors showed that by controlling the PLAL synthesis conditions and the resulting structural/chemical composition of the hybrids, one can obtain the nanomaterial with selective sensitivity toward a certain organic vapor. Designed selective chemiresistive gas sensors (also referred to as “electronic nose”) demonstrated high sensitivity up to 50 ppm and fast response/recovery time, as well as room-temperature operation modality. In another related work, Shabalina et al. reported an interesting approach to the PLAL synthesis of nanomaterials containing different phases of practically relevant bismuth silicates involving the generation of Bi and Si colloids through ablation of related bulk targets in water [3]. The authors demonstrated that subsequent laser modification of the composite Bi-Si-O nanoparticles in water results in improved catalytic activity of the resulting product with respect to the decomposition of phenol and Rhodamine B driven by soft UV light. Importantly, their work follows the recent trend of using laser post-treatment for defect engineering in PLAL-synthesized nanomaterials, allowing one to boost the catalytic activity of the product. Finally, Sandzhieva et al. reported the use of laser synthesis of Si nanoparticles in different liquids for photovoltaic applications [4]. Notably, the utilization of non-plasmonic nanoparticles supporting so-called Mie resonances is an actively developing trend in designing advanced optoelectronic devices such as thin-film photodetectors and solar cells. In their work, the authors showed that PLAL-synthesized Mie-resonant Si nanoparticles can be integrated into different functional layers of the organic solar cell, resulting in boosting the overall device efficiency from 6 to 7.5%. Overall, the collected papers related to PLAP synthesis of functional nanomaterials demonstrate the rapid development of this technology toward practically relevant applications covering catalysis, theranostics, optoelectronics and sensing.

Laser radiation of appropriate fluence above the ablation threshold of a certain material can cause morphological, chemical and phase modifications at the interface. In turn, these modifications control diverse material properties such as reflectivity, wettability, tribological performance, catalytic activity, etc. Direct laser nanotexturing of materials represents a fast growing research direction stimulated by both an increase in the fundamental understanding of laser–matter interaction phenomena as well as the rapid growth of the laser market toward the development of stable, inexpensive and high-repetition-rate pulsed sources and beam positioning systems [23]. Direct ns-laser nanotexturing of copper surfaces followed by surface hydrophobization by self-assembled monolayers of fluorinated silane was carried out by Moze et al. [8]. The authors produced several types of surface morphologies through direct laser patterning of copper sheets and evaluated their boiling heat transfer performance. For all laser-textured and chemically functionalized surfaces, a significant increase in both the critical heat flux and heat transfer coefficient was observed as compared to pristine copper surfaces. Fatkullin et al. utilized direct laser exposure of thin silver (Ag) film deposited on a flexible polymer substrate to produce hierarchical plasmon-active nanomorphology suitable for optical detection of molecular species via the surface-enhanced Raman scattering (SERS) effect [7]. The excellent SERS performance of the sensor substrate was further evaluated by detecting structural transformations of 4-nitrobenzenethiol molecular probes reacting to the changes in the cancer cell media. Importantly, the reported results bridge the gap between SERS-based bio-sensing and flexible electronics via inexpensive, scalable and environmentally friendly laser nanofabrication technology. Fominski et al. reported the deposition of composite coatings made of a tungsten sulfoselenide (WSe_x_S_y_) matrix embedding tungsten (W) nanoparticles through pulse laser ablation of WSe_2_ carried out in gaseous H_2_S [5]. The PLD-fabricated coating demonstrated improved hardness and remarkable tribological characteristics even under multiple bending cycles and low temperature tests. Komlenok et al. reported the fabrication of graphene field emitters through laser-induced forward transfer of CVD-grown single-layer graphene from a donor substrate to the acceptor one [6]. The procedure is flexible regarding the choice of acceptor substrates and leaves intact the structural and emission properties of transferred flakes, thus making it suitable for laser printing graphene-based devices.

Finally, the ability to confine energy within an ultrashort laser pulse opens up new prospects for the modification of even highly transparent materials, where the laser radiation can be focused on their bulk material, causing compositional or morphological modifications. The non-linear characteristics of light absorption combined with tight focusing within a medium with a certain refractive index n allows us to achieve extremely high recording resolution opening pathways for diverse applications including micro-optics, information encryption and data storage, optical circuits, microfluidics, etc. [24,25,26,27,28]. The first study by Kudryashov et al. demonstrated the potential to inscribe the form birefringent microstructures inside nanoporous fused silica using visible- and near-IR-range fs laser pulses [9]. The observed form birefringence was related to the hierarchical multi-scale structure of the microtracks containing nanogratings, as was observed in the experimental characterization and numerical modeling. Another related study of the same group disclosed the features of laser-induced modifications in bulk lithium niobate single crystals [10]. The authors demonstrated the formation of sub-100 nm ferroelectric domains in the vicinity of the embedded microtrack produced by near-IR and visible fs-laser pulses in a weakly filamentary nanotexturing regime. The obtained results unveiled the possibility of using direct laser writing as a tool for ferroelectric nanodomain engineering in non-linear optical ferroelectrics for the development of electrically tunable optical devices.

To summarize the content of this Special Issue, the included collection of papers clearly illustrates the growing interest of the scientific community in diverse laser-assisted nanofabrication technologies as well as the evolution of such technologies toward the processing and synthesis of practically attractive composite multi-functional materials. On a concluding note, the Guest Editors would like to express their gratitude to all of the contributing authors and reviewers, as well as to the Nanomaterial Editorial staff for their professional assistance and support. 
